# Genetic Diversity Estimation and Genome-Wide Selective Sweep Analysis of the Bazhou Yak

**DOI:** 10.3390/ani15060849

**Published:** 2025-03-15

**Authors:** Baigao Yang, Hang Zhang, Xiaoyi Feng, Zhou Yu, Jianhua Cao, Yifan Niu, Pengcheng Wan, Gang Liu, Xueming Zhao

**Affiliations:** 1 Institute of Animal Sciences (IAS), Chinese Academy of Agricultural Sciences (CAAS), No. 2 Yuanmingyuan Western Road, Haidian District, Beijing 100193, China; yangbaigao915@163.com (B.Y.); zhanghang.bio@gmail.com (H.Z.); 17806257712@163.com (X.F.); moonight189@163.com (Z.Y.); jhcao0323@126.com (J.C.); 18902075350@163.com (Y.N.); 2 State Key Laboratory of Sheep Genetic Improvement and Healthy Breeding, Institute of Animal Husbandry and Veterinary Sciences, Xinjiang Academy of Agricultural and Reclamation Sciences, Shihezi 832000, China; wanpc@hotmail.com; 3 National Animal Husbandry Service, Beijing 100193, China

**Keywords:** Bazhou yak, whole-genome sequencing, genetic diversity, selection sweep

## Abstract

The Bazhou yak, a major native meat yak breed in Xinjiang, China, is renowned for its fast growth rate, strong adaptability, and particularly high intramuscular fat (IMF) content. However, limited knowledge regarding its phylogenetic history and genomic composition has hindered its long-term conservation and utilization. This study evaluated the genetic diversity, population phylogenetics, and genome-wide selective sweep analysis (GWSA) of 100 newly obtained Bazhou yaks through genome resequencing, as well as 340 public yak genomes from nine other populations on the Qinghai–Tibet Plateau. The results revealed moderate diversity, lower genomic inbreeding levels, and rapid linkage disequilibrium (LD) decay in Bazhou yaks. Principal component analysis (PCA) and phylogenetic analysis showed a clear separation of Bazhou yaks from other yak populations, indicating the Bazhou yak as an independent genetic population. Furthermore, less genetic differentiation was found between the Bazhou yak and the Huanhu yak, while ADMIXTURE analysis revealed a common ancestral lineage between Bazhou yaks and Huanhu yaks, indicating an important genetic contribution of the Qinghai yak population to Bazhou yaks. The GWSA identified a total of 833 selected genes in Bazhou yaks using the top 5% interaction windows of both parameters (*F_ST_*, *Pi* ratio, and XP-EHH). A significant number of these genes are related to fat synthesis and deposition, such as *MTOR*, *APOA1*, and *GPAT4*. In summary, this study sheds light on the phylogenetic status and distinctive genomic features of Bazhou yaks, which facilitates our understanding of the genetic basis of the IMF phenotype in Bazhou yaks.

## Simple Summary

The Bazhou yak is a distinctive meat yak breed in Xinjiang, China, renowned for its intramuscular fat (IMF) content. This study assessed the genetic diversity, population structure, and phylogenetic relationships of Bazhou yaks in comparison to nine other yak populations. The results revealed that Bazhou yaks possess substantial genetic diversity and unique genomic features that differentiate them from other yak populations. Additionally, genome-wide selection signal scanning identified a large number of genes associated with fat synthesis and deposition in Bazhou yaks. These findings advance our understanding of the genetic basis of the IMF trait in Bazhou yaks.

## 1. Introduction

The diversified breeds of indigenous yaks have become the mainstay of the pastoral economy in the Qinghai–Tibet Plateau and its surrounding areas [1]. Among these, the Bazhou yak, a unique local breed endemic to the Bortala Mongol Autonomous Prefecture of Xinjiang, is predominantly distributed in the central of Tian Shan. Historical records indicate that the Bazhou yak population numbered fewer than 200 individuals in the 1920s but has since expanded exponentially to over 180,000 by 2024 [2,3]. It is documented that Bazhou yaks were initially introduced to Hejing County in Xinjiang from Tibet by the Mongolian royal family [4]. In the late 1980s, to meet the needs of local pastoral economic development, a large number of yaks of various breeds were introduced to improve the Bazhou yak, including breeds from Jiulong in Sichuan, Datong in Qinghai, semi-wild yaks, and local wild yaks from the Altai Mountains [3].

To date, Bazhou yaks have made significant contributions to the local economy, leveraging their notable advantages such as rapid growth, strong adaptability, and high survival rates. Notably, research has demonstrated that the intramuscular fat (IMF) content in the longest dorsal muscles of adult male Bazhou yaks can reach up to 10.91% [5], a figure markedly higher than that observed in most yaks from the Tibetan Plateau. For instance, the IMF content in Huanhu yaks is 2.23% [6], Jinchuan yaks is 5.99% [7], Mawa yaks is 1.51% [8], Niangya yaks is 2.33% [9], Pali yaks is 2.06% [9], Sibu yaks is 2.98% [9], Tianzhu white yaks is 2.01% [10], and Yushu yaks is 2.65% [11]. It is widely recognized that the fat content in fresh meat is a crucial determinant of meat quality and flavor [12]; the United States Department of Agriculture deems beef with a fat content exceeding 8% to be of high quality [13]. Consequently, the exceptional IMF levels in Bazhou yaks position them as a valuable genetic resource for breeding programs aimed at improving meat quality. However, critical gaps persist in understanding the Bazhou yak’s conservation status and the genetic mechanisms underlying its economically important traits, hindering targeted utilization and sustainable development.

Since the publication of the first domestic yak genome in 2012 [14], numerous studies have delved into the phylogenetic relationships among various yak populations. For example, Qiu et al. (2015) unveiled the domestication history of yaks, dating back to approximately 7300 BC, and pinpointed candidate selected genes associated with domestication, such as *ADCYAP1R1*, *PLXNB1*, and *SCRIB* [15]. Subsequently, Zhang et al. (2016) reported on the genetic differentiation and copy number variation (CNV) distribution patterns between wild yaks and the domestic Tibetan Plateau yaks [16]. Wang et al. (2019) further contributed a CNV map of 16 yak populations and identified a suite of candidate genes associated with hypoxic adaptation, including *DCC*, *MRPS28,* and *GSTCD* [17]. Moreover, studies have confirmed the association of *TUBA8* and *TUBA4A* with the multi-ribbed traits of Jinchuan yaks through comparative whole-genome selection analysis [18,19]. Liu et al. (2023) identified structural variant (SV) divergence patterns between wild and domestic yaks, elucidating that an SV in the KIT gene serves as a critical genetic determinant for the white coat phenotype in yaks [20]. Wu et al. (2024) utilized whole-genome sequencing data to identify several candidate genes related to the brown coat phenotype in yaks, such as *PLCB1*, *LEF1*, and *DTNBP1* [21]. Additionally, Peng et al. (2024) utilized genome-wide data to reveal genes associated with yak domestication, including *ANKRD28*, *HECW1*, and *HECW2* [22]. Gangwar et al. (2024) uncovered genomic differences among Indian, Chinese, and wild yak populations, identifying species-specific marker genes like *ADGRB3*, *ANK1*, and *CACNG7* [23].

Meat quality in yaks is closely tied to lipid metabolism and fat deposition. For example, studies have shown that the differences in meat tenderness between female and male yaks’ longissimus dorsi muscle are related to the distribution of fatty acids, which may be related to the expression levels of some genes, such as *SCD*, *PLIN5*, and *LPL* [24]. Subsequently, a study used lipidomics and RNA sequencing technology to reveal the relationship between fat content and gene expression in the longissimus dorsi muscle of yaks and pointed out key candidate genes related to muscle fatty acid content, including *FASN*, *SLC16A13,* and *FADS6* [25]. In 2023, Luo et al.’s study based on the whole transcriptome of the longissimus dorsi muscle of yaks demonstrated that HIF-1α is a key regulator of fat content, controlling fat deposition in yak adipocytes by regulating the expression of C/EBPα and *FABP4* [26]. Moreover, Ding et al. (2024) reported differences in the expression of lipid metabolism-related genes in yak adipose tissue under different feeding conditions, including *ACACA*, *INSIG1*, and *ACSL1* [27]. Furthermore, Xu et al. (2024) utilized yak adipose tissue RNA sequencing data and proteomics data to reveal candidate genes related to yak fat deposition, including *MYL3*, *ACADS*, *L2HGDH*, etc., among which *ACADS* plays a vital role in the negative feedback regulation of fat deposition [28]. These studies provide important references for research on the meat quality traits of yaks.

However, to date, no research has been conducted on the genome-wide analysis of Bazhou yaks and a comprehensive understanding of the genomic genetic basis of IMF traits in Bazhou yaks is still lacking. This study employs whole-genome sequencing to assess genetic diversity and population structure of Bazhou yaks and identify candidate genes associated with high IMF content through genome-wide selection scans. Our findings aim to inform conservation strategies for Bazhou yaks and advance the genetic understanding of economically important meat trait in yak breeds.

## 2. Materials and Methods

### 2.1. Sample Collection and Sequencing

In this study, blood samples from 100 Bazhou yaks (Bazhou) were provided by a farm containing more than 6000 Bazhou yaks in Hejing County, Xinjiang, China (altitude > 3000 m, N 42°19′19.1″, E 86°22′54.3″). All individuals were confirmed to have no kinship within three generations. Genomic DNA was extracted using the TIANamp Genomic DNA Kit (TIANGEN, China). Sequencing libraries were prepared with the Annoroad^®^ Universal DNA Library Prep Kit v2.0 (Illumina^®^, San Diego, CA, USA), and sequenced on the BGISEQ-500 platform (Beijing Genomics Institute, Shenzhen, China) with an average coverage depth of 10× per sample.

Additionally, 340 publicly available yak genome datasets from nine populations were downloaded from the Sequence Read Archive (SRA) database (Table S1). These populations included Huanhu yaks (n = 34), Jinchuan yaks (n = 149), Maiwa yaks (n = 31), Niangya yaks (n = 22), Pali yaks (n = 10), Sibu yaks (n = 5), Tianzhu white yaks (n = 40), Yushu yaks (n = 27), and Wild yaks (n = 22), which were subsequently utilized in population evolution and selective sweep analysis.

### 2.2. SNP Calling and Annotation

Raw sequencing data (15,056.99 GB) were filtered using fastp (v0.23.4) [29] with default parameters, yielding 14,364.80 GB of clean data (99.38 GB clean reads). Clean reads were aligned to the yak reference genome (NWIPB_DYAK_1.0, GCA_027580245.1) using bwa-mem2 (v2.2.1) [30]. SNP calling was performed via the HaplotypeCaller module of GATK (v4.5.0.0) [31] with a joint calling method. No reference SNP correction was applied to preserve breed-specific variants, followed by hard-filtering with the parameters “QD < 2.0, QUAL < 30.0, SOR > 3.0, FS > 60.0, MQ < 40.0, MQRankSum < −12.5, ReadPosRankSum < −8.0”. Filtered SNPs were further processed using PLINK 2.0 (www.cog-genomics.org/plink/2.0/, accessed on 13 March 2025) with the parameters “--min-alleles 2 --max-alleles 2 –not-chr X --geno 0.1 --maf 0.05”, retaining 11,403,916 high-quality autosomal biallelic SNPs. Functional annotation of the SNPs was conducted using the Annovar software (v2024Feb19) [32].

### 2.3. Genetic Diversity Estimation and Phylogenetic Analysis

The observed heterozygosity (*Ho*) and expected heterozygosity (*He*) for each population were calculated using PLINK (v1.9) [33]. The nucleotide diversity (*Pi*) was estimated using vcftools (v0.1.16) [34] with a sliding window size of 50 kb and a step size of 20 kb. Linkage disequilibrium (LD) for each population was assessed via PopLDdecay (v3.43) [35]. The runs of homozygosity (ROH) were detected through Plink (v1.9) [33] using the following parameters: a window size of 50 kb, a maximum of three heterozygous SNPs, a minimum SNP density of 50, a minimum homozygosity percentage of 5%, and a gap size of 100 kb between windows [21,36]. ROH regions longer than 300 kb were considered. The inbreeding coefficients based on ROH (FROH) were calculated using the following formula:

$$FROH=\frac{\sum LROH}{Lauto}$$

where ∑LROH means the total length of ROHs of each individual, Lauto means the total length of the autosomal genome (2432.055 Mb, consistent with the NWIPB_DYAK_1.0 genome assembly).

Additionally, SNP sites with high LD were filtered by plink (v1.9) [33] with the following parameters “--indep-pairwise 50 10 0.2”, and 991,997 remaining SNPs were used for principal component analysis (PCA), population structure analysis and phylogenetic tree construction. The PCA was performed by plink (v1.9) [33] and visualized via ggplot2 (v3.5.1) [37]. Population clustering analysis was performed using ADMIXTURE (v1.3.0) [38,39], considering 2 to 10 clusters (*K*). The results for *K* = 2 to 5 were visualized using Pophelper (v2.3.1) [40]. The maximum likelihood (ML) phylogenetic tree was constructed using the TVM + F+I + R10 model in iqtree2 (v 2.3.6) [41] and visualized using ggtree (v3.14.0) [42]. The pairwise population genetic distance was calculated via vcftools (v0.1.16) [34] with the parameters “--fst-window-size 50000 --fst-window-step 20000”, and a neighbor-joining (NJ) tree was generated in PHYLIP (v3.6) (https://evolution.gs.washington.edu/phylip.html, accessed on 1 January 2025) and visualized using iTOL (v6) [43].

### 2.4. Selective Sweep Analysis

The genome-wide selective sweep analysis (GWSA) between the Bazhou yak and the other eight domestic yak populations (Huanhu yaks, Jinchuan yaks, Maiwa yaks, Niangya yaks, Pali yaks, Sibu yaks, Tianzhu white yaks, and Yushu yaks) was performed using three methods: the fixation index (*F_ST_*) [44], the differences in nucleotide diversity (*Pi* ratio) [45], and the cross-population extended haplotype homozygosity (XP-EHH) [46]. *F_ST_* and *Pi* ratio analysis were performed using vcftools (v0.1.16) [34] with a 50 kb sliding window and a 20 kb step [39]. For XP-EHH analysis, firstly, haplotype phasing for each population was performed using Beagle (v5.4) [47], followed by calculation of XP-EHH using Selscan (v2.0.3) [48]. The average normalized XP-EHH scores for 50 kb regions were used to identify candidate selective sweep regions. The top 5% windows of each method were extracted, and interaction regions of at least two methods were considered as candidate selective regions, with the following thresholds: *F_S_* ≥ 0.0604692, *Pi* ratio ≥ 1.31258, and XP-EHH ≥ 1.321455. Candidate selected genes located in overlapping regions were obtained using bedtools (v2.31.1) [49].

### 2.5. Gene Function Annotation

The Gene Ontology (GO) and the Kyoto Encyclopedia of Genes and Genomes (KEGG) enrichment of selective genes were performed using KOBAS (v2.0) [50] (accessed on 10 January 2025); the significance threshold of pathway enrichment was considered as *p* < 0.05.

## 3. Results and Discussion

A total of 3424.44 GB raw sequencing data were generated from 100 Bazhou yaks. After rigorous quality control measures, 3242.45 GB of high-quality data were retained for alignment against the reference genome (Table S2). Subsequent variant annotation led to the identification of 11.40 million autosomal SNPs across all 440 yak individuals. These SNPs were predominantly located within intergenic regions (accounting for 74.73%) and intronic regions (comprising 23.58%). Within exonic regions, a detailed annotation revealed 36,976 synonymous variants and 25,813 nonsynonymous variants (Table S3).

**Figure 1:**
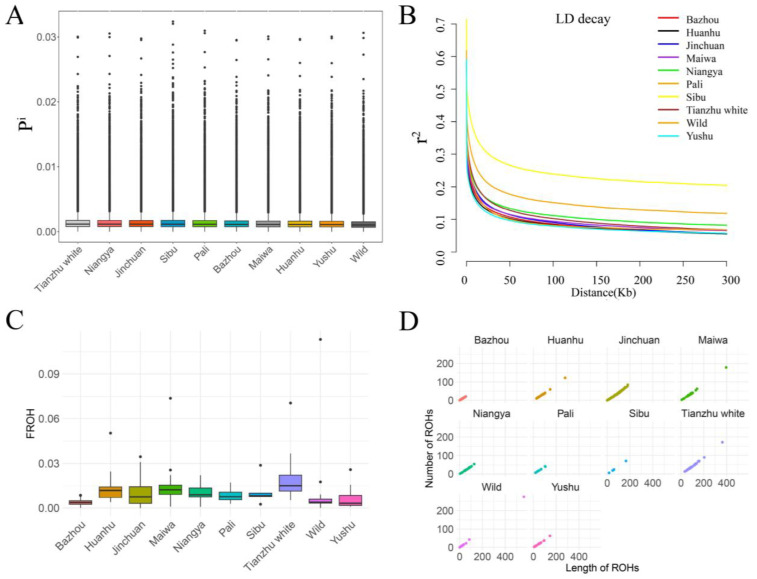
The genetic diversity of the Bazhou yak and the other nine yak populations. (**A**) The genome-wide distribution of nucleotide diversity of each population. The black line in the boxplot is the median line and the outside points are outliers. (**B**) Genome-wide average LD decay is estimated from each population. One color line represents one population. (**C**) Number of ROHs and the total ROH length of each yak individual. (**D**) Box plot of the genomic inbreeding for each population.

Genetic diversity analysis revealed that the *He* across all populations ranged from 0.264983 to 0.299342. Specifically, the *He* of the Bazhou yak was 0.293166, which was higher than its *Ho* of 0.259915 (Table S4). Moreover, similar levels of *Pi* were observed among the 10 yak populations, spanning a range of 0.001212 to 0.001391, with the Bazhou yak displaying a moderate level of *Pi* at 0.001337 (Figure 1A, Table S4). Furthermore, LD decay analysis demonstrated that the Yushu yak exhibited the most rapid decay of LD, a pattern mirrored in geographically isolated populations, including Huanhu yaks, Bazhou yaks, and Maiwa yaks (Figure 1B).

In the context of ROH analysis, a comprehensive total of 24,871 ROH segments were detected across the 10 yak populations, with fragment lengths varying between 300 Kb and 2 Mb (Figure 1C, Table S5). Notably, the Bazhou yak population had the shortest and least cumulative length of ROH segments. Moreover, the FROH among all yak populations ranged from 0.003 to 0.018, with the Bazhou yak population exhibiting the lowest FROH value of 0.003 (Figure 1D). These findings suggest that despite the Bazhou yak population’s initially limited size, its bloodline has been continuously enriched through multiple introductions over recent decades (Li et al., 2017). Additionally, the adoption of free-grazing practices has helped avoid the pitfalls of intensive production models that could potentially disrupt the animals’ mating system. Consequently, due to random mating, the integration of exogenous bloodlines, and population expansion, the Bazhou yak population has maintained a rich genetic diversity and a relatively low level of genomic inbreeding.

**Figure 2:**
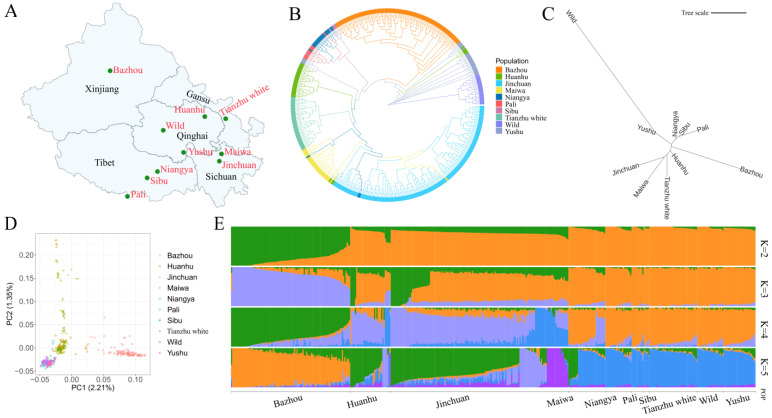
Population structure of the Bazhou yak and its relationship with the other nine yak populations. (**A**) Geographic distribution of 10 yak populations in China. (**B**) ML tree of 440 yaks. (**C**) NJ tree based on population pairwise *F_ST_* value among 10 yak populations. (**D**) PCA plot of 440 yaks. Different colored points represent different populations. (**E**) Model-based clustering of different yak populations using ADMIXTURE with K ranging from 2 to 5. Population names are at the bottom of the plot.

The individual ML-tree (Figure 2B) reveals that all Bazhou yaks cluster together show no admixture with other populations. Additionally, similar phylogenetic relationships were found between the three Tibetan yak populations (Niangya yaks, Pali yaks, and Sibu yaks), which was consistent with the previous report by Ji et al. (2021) [51]. According to the *F_ST_*-based NJ tree presented in Figure 2C, the Bazhou yak is genetically closest to the Qinghai Huanhu yak and farthest from the Wild yak (Table S6). The PCA reveals that the first principal component (PC1, accounting for 2.21%) distinctly separates most Bazhou yaks from other yak populations, while the second principal component (PC2, accounting for 1.35%) primarily separates Bazhou yaks, Huanhu yaks, and Jinchuan yaks into the same group (Figure 2D). The ADMIXTURE analysis indicates that at *K* = 2, all yak individuals exhibit evidence of multi-lineage ancestry (Figure 2E). When *K* = 3, Bazhou yaks, Huanhu yaks, and Jinchuan yaks further diverge into two distinct lineages. At *K* = 4, which corresponds to the minimum cross-validation (CV) error, some Bazhou yaks exhibit a unique lineage, while others display a mixed lineage resembling that of Huanhu yaks (Table S7). Notably, the geographic distribution of Bazhou yaks is in the Tianshan Mountains, isolated from other domestic yak populations on the Qinghai–Tibet Plateau, as shown in Figure 2A. Both the ML tree and PCA results suggest that the Bazhou yak has evolved into a unique genetic population. Furthermore, compared to other yak populations, the Bazhou yak exhibits a closer genetic distance to the Huanhu yak (Figure 2B). Additionally, ADMIXTURE results reveal that Bazhou yaks share a common ancestral lineage with some Huanhu yaks, suggesting that the Qinghai yak population had important genetic contributions to the Bazhou yak, which is consistent with the view proposed by Li et al. (2017) [3]. In summary, despite the influence of external genetic factors, Bazhou yaks have gradually evolved into a distinct local population through natural and artificial selection over a century, distinguishing it from other yak populations.

**Figure 3:**
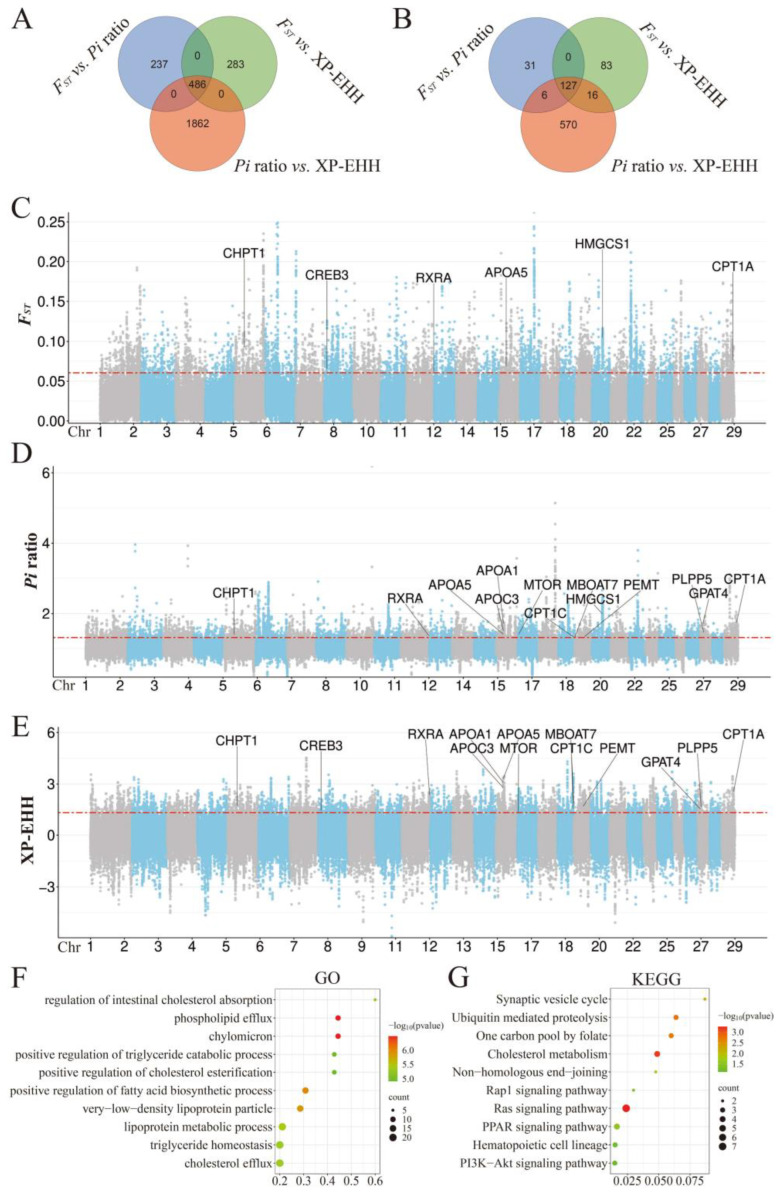
Results of selective sweep analysis between the Bazhou yak and eight other domestic yak populations. (**A**) Selective regions detected by pairwise combinations of *F_ST_*, *Pi*, and XP-EHH methods. (**B**) Candidate-selected genes obtained from the selective regions. (**C**) Manhattan plot of the genome-wide distribution of *F_ST_* value. (**D**) Manhattan plot of the genome-wide distribution of *Pi* ratio value. (**E**) Manhattan plot of the genome-wide distribution of XP-EHH value. (**F**) GO terms enriched by candidate selected genes. (**G**) KEGG pathways enriched by candidate selected genes.

The GWSA identified a total of 164 candidate genes (CDGs) within 723 genomic overlapping regions (GORs) using the *F_ST_* and *Pi* ratio methods (Figure 3A–C, Table S8), 226 CDGs within 769 GORs using the *F_ST_* and XP-EHH methods (Figure 3A,B,D, Table S9), and 719 CDGs within 2348 GORs using the *Pi* ratio and XP-EHH methods (Figure 3A,B,E, Table S10), respectively, with the top 5% interaction window thresholds set at *F_ST_* ≥ 0.0604692, *Pi* ratio ≥ 1.31258, and XP-EHH ≥ 1.321455. Among the total of 833 CDGs located in these genomic overlapping regions, 767 of them were found to be significantly enriched in 549 GO terms (Figure 3E, Table S11) and 92 KEGG pathways (Figure 3F, Table S12). Notably, the majority of these genes exhibited significant enrichment in metabolic pathways, including *B4GALNT4*, *ATP6V1D*, and *GGCX*. Additionally, they were enriched in the PI3K-Akt signaling pathway (e.g., *PIK3CD*, *MTOR*, and *RPTOR*) and the MAPK signaling pathway (e.g., *MAP2K1*, *MAP4K3*, and *NLK*). Remarkably, numerous genes were distinctly involved in pathways related to fatty acid biosynthesis, such as the PPAR signaling pathway (e.g., *RXRA*, *HMGCS1*, and *APOA1*), glycerophospholipid metabolism (e.g., *GPAT4*, *PLPP5,* and *MBOAT7*), and the adipocytokine signaling pathway (e.g., *CPT1A*, *CPT1C*, and *G6PC2*).

It is well documented that the IMF content in the muscles of domesticated meat animals is closely related to the biological mechanisms that control fat synthesis and deposition [52]. Our study has drawn particular attention to the abundance of selected genes in Bazhou yaks that are associated with lipid synthesis and metabolism. For example, *PIK3CD* and *mTOR* are enriched in pathways that mediate insulin-regulated de novo lipogenesis, which is crucial for fat synthesis in adipose tissue [53]. *PIK3CD*, a class I phosphoinositide 3-kinase (PI3K) member, plays a pivotal role in downstream Akt activation [54]. Extensive research underscores the indispensable function of the PI3K/Akt pathway in insulin-mediated de novo lipogenesis [55]. In yaks, recent studies have demonstrated a connection between the PI3K/Akt pathway and fat deposition in the longissimus dorsi muscle [56]. Qin et al. (2024) further reported that miR-129 enhances the activation of the PI3K/Akt pathway in intramuscular adipocytes, thereby promoting the differentiation and adipogenesis of intramuscular preadipocytes. In pigs, a positive correlation has been observed between *PIK3CD* expression in the longissimus dorsi muscle and intramuscular fat content [57]. Moreover, mTOR, a downstream effector of the PI3K/Akt pathway, is integral to the regulation of fat synthesis [58]. The proteins encoded by *mTOR* and *RPTOR* constitute essential subunits of mTORC1, which modulate fat synthesis via S6K1, SREBP, and lipin [59]. Additionally, mTOR serves as a core component of mTORC2, another vital regulator of fat synthesis [60]. Studies in Caenorhabditis elegans have shown that mTORC2 regulates fat storage through SGK-1 [61], while mTORC2 deficiency in brown mouse adipose tissue impairs fat generation [62]. These findings collectively emphasize the significance of the PI3K/Akt/mTOR pathway in the regulation of IMF content in domesticated meat animals.

Numerous studies have revealed the key regulatory role of the PPAR signaling pathway in fatty acid metabolism [63]. Notably, several CDGs are significantly enriched in this pathway, including *RXRA*, *HMGCS1*, *APOA1*, *APOA5*, and *APOC3*. *RXRA*, a member of the retinoid X receptor family, acts as a pivotal heterodimerization partner for PPARγ in adipose tissue, mediating lipid transport, storage, and metabolism [64]. Elevated *RXRA* expression levels have been observed in the fat tissue of obese chickens compared to lean ones [65]. Similarly, high *RXRA* expression is noted in duck preadipocytes, facilitating the accumulation of triglycerides in adipocytes [66]. Significantly, Qian et al. reported that *RXRA* promoted the differentiation of mesenchymal stem cells into adipocytes [67], confirming its significant contribution to fat deposition in adipose tissue. In parallel, *HMGCS1*, a key enzyme in cholesterol biosynthesis, catalyzes the formation of HMG-CoA, a precursor to cholesterol [68]. Previous research has demonstrated a positive correlation between cholesterol content and fat content in commercial pig adipose tissue [69]. *HMGCS1*’s involvement in lipid metabolism is further supported by studies showing that its overexpression increases total cholesterol, triglycerides, and lipid droplets in ACSS2 knockout neoplasm cells [70]. Furthermore, *APOA1* encodes the apolipoprotein A-I, a major component of high-density lipoprotein that facilitates de novo synthesis of cholesterol esters and triglycerides [71]. Another apolipoprotein, *APOA5*, regulates plasma triglyceride levels, and mutations in *APOA5* lead to increased blood triglycerides and fat deposition [72]. Similarly, *APOC3* modulates lipid metabolism by inhibiting low-density lipoprotein-mediated lipolysis and hepatic lipase-mediated conversion of very low-density lipoprotein, thereby influencing blood triglyceride levels and promoting hepatic fat accumulation [73]. Notably, *APOC3* genetic knockout enhances low-density lipoprotein dependent triglyceride-derived fatty acid uptake and fat deposition in mouse adipose tissue [74].

Rich findings determinate that chemical modification of fatty acids provides essential substrate sources for fat synthesis [75], and *GPAT4*, *PLPP5*, *CHPT1*, *PEMT*, and *MBOAT7* have been found to be involved in lipid metabolic processes such as dephosphorylation of fatty acids, lipid esterification, and acyl transfer. *GPAT4*, a rate-limiting enzyme in triglyceride biosynthesis, exhibits high expression levels in both brown and white adipose tissues [76]. This enzyme catalyzes the conversion of glycerol-3-phosphate and acyl-CoA into lysophosphatidic acid, which subsequently reacts with acyl-CoA to produce phosphatidic acid [77]. Phosphatidic acid is then dephosphorylated by phospholipid phosphatases to generate diacylglycerol (DAG) [78]. Consistent with its functional importance, *GPAT4*-deficient mice exhibit reduced adipose triglyceride accumulation, diminished white adipocytes, lower DAG and triacylglycerol levels, and altered monounsaturated/polyunsaturated fatty acid profiles [79]. Of particular interest, *PLPP5* (a phosphatidic acid phosphatase family member) catalyzes the dephosphorylation of lysophosphatidic acid, PA, and diacylglycerol pyrophosphate to produce DAG and inorganic phosphate, thereby facilitating triglyceride biosynthesis [78]. Additionally, *CHPT1* encodes choline phosphotransferase, an enzyme that catalyzes the synthesis of phosphatidylcholine (PC) from phosphorylcholine and DAG [80]. PC is essential for fat absorption and transport in animals [81], and polymorphisms in the *CHPT1* gene have been significantly associated with fat deposition traits in pigs [82]. Moreover, *PEMT* facilitates hepatic PC synthesis via phosphatidylethanolamine methylation, serving dual roles in both de novo PC generation and lipoprotein-mediated fat biosynthesis [83]. Mechanistically, *PEMT* orchestrates adipocyte differentiation and adipogenesis through ERK1 and Akt signaling pathways [84]. Furthermore, *MBOAT7*, a lysophosphatidylinositol acyltransferase, regulates liver fat deposition, and its knockout leads to excessive liver fat accumulation [85]. *MBOAT7* also mediates the remodeling of arachidonic acid-containing phosphatidylinositol [86]. Arachidonic acid is a significant flavor compound in meat, and *MBOAT7* serves as the primary source of arachidonic acid-containing phosphatidylinositol in adipose tissue [87].

The degree of fat deposition is further influenced by the regulation of fatty acid oxidation (FAO) rate-controlling genes [88]. We found that some selected candidate genes (*CREB3*, *CPT1A*, and *CPT1C)* are enriched in the FAO process. *CREB3* belongs to the leucine zipper family of DNA-binding proteins and plays a pivotal role in modulating lipid oxidation and triglyceride storage through its mediation of *PPARGC1A* transcriptional regulation [89,90]. In the context of lipid storage maintenance, *CREB3* holds a crucial position, as evidenced by *CREB3* knockout mice exhibiting heightened PGC-1α expression, elevated energy expenditure, enhanced gluconeogenesis, and reduced body fat levels [91]. Moreover, *CPT1A*, a carrier protein tasked with transporting fatty acids into mitochondria and regulated by acetyl-CoA carboxylase 1, a key enzyme in fatty acid synthesis, also plays a significant role in the FAO process [92]. Studies indicate that overexpressing *CPT1A* promotes the proliferation of goat intramuscular preadipocytes, facilitating the subsequent development of adipose tissue [93]. Another fatty acid transporter, *CPT1C*, exerts an influence on triglyceride content in both liver and intramuscular tissues. Research has demonstrated that *CPT1C* knockout mice are more prone to high-fat diet-induced intramuscular triglyceride accumulation [94]. In summary, these findings enhance our understanding of the genetic mechanisms underlying IMF deposition in Bazhou yaks and provide a theoretical foundation for targeted breeding strategies and genetic marker development aimed at meat quality improvement.

## 4. Conclusions

This study comprehensively investigated the genomic variations in the Bazhou yak using whole genome sequencing technology, revealing its population’s genetic diversity and confirming the formation of a distinct genetic structure significantly different from other Qinghai Tibetan plateau yak populations. These results provide foundational data and strategic guidance for the genetic assessment, resource conservation, and sustainable utilization of the Bazhou yak. Additionally, selective sweep analysis uncovered candidate genes potentially influencing IMF-related meat quality traits, offering novel insights into genomic selection markers for Bazhou yaks. These findings advance our understanding of the genetic basis underlying yak meat quality traits and provide important directions for formulating rational breeding strategies in future genetic improvement programs.

## Data Availability

The sequencing data used for analysis have been submitted to China National GeneBank DataBase database with project number CNP0005893. In addition, public data were downloaded from the SRA database with project numbers PRJNA285834, PRJNA483376, PRJNA508860, PRJNA540974, PRJNA670822, PRJNA766811, PRJNA899924, and PRJNA950586.

## Supplementary Materials

The following supporting information can be downloaded at: https://www.mdpi.com/article/10.3390/ani15060849/s1, Table S1. Information on yak data downloaded from the SRA database. Table S2. Sequencing information of 100 Bazhou yaks. Table S3. Information on autosomal genomic region variants annotation. Table S4. The genetic diversity of the 10 yak populations. Table S5. Numbers of ROH and genomic inbreeding (FROH) of the 10 yak populations. Table S6. Pairwise population *F_ST_* values. Table S7. Cross validation (CV) errors generated by ADMIXTURE at cluster (K) ranging from 1 to 10. Table S8. Selective regions detected by *F_ST_* and *Pi* ratio methods in the Bazhou yak. Table S9. Selective regions detected by *F_ST_* and XP-EHH methods in the Bazhou yak. Table S10. Selective regions detected by *Pi* ratio and XP-EHH methods in the Bazhou yak. Table S11. The result of the Gene Ontology (GO) analysis. Table S12. The result of Kyoto Encyclopedia of Genes and Genomes (KEGG) analysis.

## Abbreviations

The following abbreviations are used in this manuscript:

CDG	Candidate genes
CNV	Copy number variation
DAG	Diacylglycero
FAO	Fatty acid oxidation
FST	The fixation index
GO	Gene Ontology
GORs	Genomic overlapping regions
GWSA	Genome-wide selective sweep analysis
He	The expected heterozygosity
Ho	The observed heterozygosity
IMF	Intramuscular fat
KEGG	Kyoto Encyclopedia of Genes and Genomes
LD	Linkage disequilibrium
ML	Maximum likelihood
NJ	Neighbor-joining
PC	Phosphatidylcholine
PCA	Principal component analysis
Pi	The nucleotide diversity
PI3K	Phosphoinositide 3-kinase
ROH	The runs of homozygosity
XP-EHH	The cross-population extended haplotype homozygosity
